# In this month’s Bulletin

**DOI:** 10.2471/BLT.23.000523

**Published:** 2023-05-01

In the editorial section, Ahmad Reza Hosseinpoor et al. (298) present WHO's Health Inequality Data Repository. Ritu Sadana et al. (299) call for papers for an upcoming theme issue on building an economy for health. Rosamund F Lewis et al. (300) list the latest recommendations for countries responding to mpox. 

Tatum Anderson (303–304) reports on the requirements for developing an effective tuberculosis vaccine. Vikram Patel talks to Gary Humphreys (305–306) about developing evidence-based approaches to collaborative mental health service provision.

## Shaping global research and development

Sarah C Charnaud et al. (326–330) introduce a new feature in the Bulletin: WHO target product profiles.

Wallace White et al. (331–340) provide specifications for new readers of rapid diagnostic tests.

Suman Rao et al. (341–345) detail the aerosolized surfactants needed to treat neonatal respiratory distress.

Matthias Helble et al. (355–357) describes WHO’s new coordinated scientific advice procedure and other services provided to product developers.

## India

### Preventing polio transmission

Madhu Chhanda Mohanty et al. (346–354) establish a surveillance network for patients with primary immunodeficiencies.

## China 

### Early life and healthy ageing

Yafei Si et al. (307–316) investigate how early-life factors are associated with intrinsic capacity.

### Over the counter antibiotics

Jie Chen et al. (317–325) track sales by pharmacies in the absence of prescriptions.

## Global

### Beyond baby formula

Elizabeth K Dunford & Barry M Popkin (358–360) describe global supply of, and demand for, ultraprocessed foods for children.

